# Mental health stigma and mental health knowledge in Chinese population: a cross-sectional study

**DOI:** 10.1186/s12888-020-02705-x

**Published:** 2020-06-22

**Authors:** Huifang Yin, Klaas J. Wardenaar, Guangming Xu, Hongjun Tian, Robert A. Schoevers

**Affiliations:** 1 Tianjin Mental Health Institute, Tianjin Anding Hospital, No. 13, Liulin Road, Hexi District, Tianjin, 300222 China; 2 Department of Psychiatry, Interdisciplinary Center Psychopathology and Emotion regulation (ICPE), University of Groningen, University Medical Center Groningen, Hanzeplein 1, 9713 GZ Groningen, The Netherlands

**Keywords:** China, stigma, Mental health knowledge, Survey

## Abstract

**Background:**

Little is known about the public stigma on mental illness and mental health knowledge (MHK) in China, public stigma and low MHK can negatively affect patients’ health and increase the burden of mental disorders on society. This study aimed at investigating the rates of stigma and MHK, the correlates of stigma and MHK, and the association between MHK and stigma among a Chinese population.

**Methods:**

The data is from the Tianjin Mental Health Survey (TJMHS), which involved a large and a representative sample of adult community residents in the Chinese municipality of Tianjin (*n* = 11,748). In a 12% random subsample (*n* = 1775) the Perceived Discrimination and Devaluation scale (PDD) and a Mental Health Knowledge Questionnaire (MHKQ) were administered. First, percentages of the responses to the individual items of the PDD and MHKQ were investigated. Second, sociodemographic correlates of PDD and MHK, and the association between stigma and MHK were investigated.

**Results:**

We found that a sizable proportion of participants responded that others would hold a negative attitude towards (former) mental patients, especially with regard to engaging in closer personal relationships. Most people were not familiar about the causes, treatments and prevention of mental illness. Resident area, age, education level, Per capita family income and employment status were related to devaluation score and MHKQ score. MHK was negatively associated with public stigma.

**Conclusions:**

There is room for improvement with regard to levels of public stigma and MHK in China. Providing psychoeducation to improve public MHK could also contribute to reduction of public stigma.

## Background

The public stigma on mental illnesses manifests itself in the way the population reacts to mental [1, 2]. Public stigma have negative effects on the lives of people with mental illness, by preventing them from pursuing vocational, housing, and healthcare goals, and holding them back from seeking treatment [3, 4] and affecting the quality of delivered healthcare [5]. The Global Context–Mental Health Study has shown public hold high prejudice attitude to patients with mental illness in 16 countries [6]. Therefore, researchers and policy-makers have sought to develop strategies to destigmatize mental illness. One suggested strategy has been to increase mental health knowledge (MHK) with education programs. Indeed, a range of studies have shown that brief courses about mental illness may reduce prejudices and stigmatizing attitudes among a wide variety of participants [7, 8]. In China, it has been proposed that mental health stigma in China might be addressed by increasing MHK, using national education programs [9]. However, the number of investigations of mental-health stigma and MHK, and their interrelatedness, has been limited, making it hard to judge if and how educational programs should be targeted. Previous work about stigma on mental illness in china has indicated that public stigma is indeed perceived by patients and their families [10, 11]. In addition, several surveys have shown high public stigma [12–14] and low MHK about depression [15, 16] and schizophrenia [17, 18]. Finally, recent research has raised concerns that mental health professionals hold discriminatory attitudes towards psychiatric patients in China [19]. However, an important research question concerns the actual relationship between MHK and public stigma. In addition, different aspects and/or types of stigma (devaluation vs. discrimination) and their relationships to MHK need to be further investigated. Finally, the role of sociodemographic characteristics (e.g. gender, age, education) and mental health status need to be considered when investigating stigma and its relationship to MHK.

To this end, stigma and MHK were both measured in a large and representative sub-sample (n = 1775), drawn from the Tianjin Mental Health Survey (TJMHS) [20]. Tianjin is a municipality that has seen rapid socioeconomic development (e.g., migration, urbanization) and, as such, is a typical example of the rapid changes that occur in many regions of China. Therefore, results from the current study could also give an indication of the prevalence and interrelatedness of stigma and MHK in other regions in China, for which up-to-date data on mental illness stigma and MHK are currently scarce. This study aimed to investigate: (1) the rates of stigma and MHK, (2) the associations of stigma and MHK with sociodemographic characteristics, and (3) the association between MHK and stigma.

## Methods

### Sample and procedures

Tianjin, one of the four municipalities directly under the central government, is also the largest port city and industrial and commercial city in northern China. It is located in the northeast of the North China Plain, north of Beijing in and lies at the coast of the Bohai Sea. By the end of 2018, Tianjin had a permanent population of 15.60 million with a per capita gross domestic product (GDP) of Ұ120,711 [21]. Data came from the TJMHS (*n* = 11,748), which was conducted between July 2011 and March 2012. A detailed description of the survey design, methods and objectives can be found elsewhere [20]. In short, the TJMHS used a two-phase design to include a large, representative community sample of respondents aged 18 and older in the Tianjin region. Participants were selected with a multistage cluster random sampling method, and were selected using probability proportionate to size of the population of each primary sampling unit. Initially, a total of 11,748 subjects were screened for psychopathology risk.

Of the total sample, a random 12% was selected and was administered additional questionnaires about stigma and MHK. We calculated the number of subjects that needed to complete the MHKQ to achieve sufficient statistical power based on the formula *n* = μ_α_^2^ p (1 - p)/δ^2^ , where μ_α_ = the one-sided magnitude of the confidence level (at α = 0.05, μ_α_ = 1.96), p = expected proportion of the outcome of interest (we assume that 50% individuals have the correct answers of MHKQ), and δ = margin of error (δ = 50% × 0.05 = 0.025). The calculated necessary minimum sample size was 1537. The number of subjects to be approached was set 20% higher, at 1844, which comprises about 12% of the total TJMHS sample of *n* = 15,482. As part of the survey design, participants were included from randomly selected households located in primary sampling units (villages or neighborhoods). All households within each PSU were assigned a unique ID number (1000, 1001, 1002, 1003 etc.) and only participants with ID numbers ending with the numbers 00 to 11 were selected into the subsample, which contained roughly 12% of all subjects. Of the initially selected subsample (*n* = 1775), 130 persons refused to participate and 30 persons only partially completed the assessment. Of the selected participants, 1609 completed the stigma questionnaire, 1615 completed the MHK questionnaire (MHKQ) and 1591 respondents completed both. The latter sample was used for the current analyses. The study protocol was approved by the medical ethics committee of the Tianjin Mental Health Center and all respondents signed informed consent prior to participation.

### Measures

Although the instruments were originally self-report questionnaires, they were interviewer-administered in the TJMHS because a considerable part of respondents were expected to be semi- or illiterate. In these versions, the interviewer read the items aloud and recorded the participant’s responses. These versions were used in all participants, including literate respondents, to ensure standardized measurements.

### The perceived discrimination and devaluation scale (PDD)

The PDD [2, 22] is a 12-item questionnaire to assess expectations of devaluation and discrimination toward current or former psychiatric patients. The items assess how “most people” or “most employers” think or act toward persons with a current or a prior psychiatric disorder. The items are rated on a five-point scale. Two subdomains are assessed in the PDD [23, 24]. Perceived *devaluation* refers to expectations about how others see (former) mentally ill persons (e.g., as being dangerous, untrustworthy). Perceived *discrimination* refers to expectations about how others will act toward (former) mentally ill persons (e.g., keeping them at a distance, denying them opportunities). The current study used the Chinese version of the PDD, which has the same items as the original but uses a slightly modified response scale, adding the option ‘not sure’. This version was previously shown to have acceptable psychometric properties [25]. Individual PDD items were also inspected. For this, each item response was categorized into three categories, coding ‘completely agree’ and ‘basically agree’ as ‘agree’, and ‘basically disagree and ‘completely disagree’ as ‘disagree’.

### Mental health knowledge questionnaire (MHKQ)

A 16-item questionnaire, developed by the Ministry of Health of China was used to assess MHK (see Table 3). The scale consists of sixteen items rated on a dichotomous response scale (yes/no). One point is given for each ‘yes’ response on items 1, 3, 5, 7, 8, 11, 12, 15, 16 and 1 point is given for each ‘no’ response on items 2, 4, 6, 9, 10, 13 and 14. The item scores are added up to a total score (range: 0–16), with higher scores indicating higher MHK.

### General health questionnaire (GHQ-12)

The Chinese version of the GHQ-12 [26] assesses general psychological distress with 12 items, rated on 4-point scale. Each item was scored as follows: 0 = ‘better than usual’, 0 = ‘as usual’, 1 = ‘less than usual’ and 1 = ‘much less than usual’, resulting in a sum score with a range of 0–12. Respondents with a GHQ-12 score above 3 were considered to have some mental problems. Previous work showed the Chinese GHQ-12 to have adequate internal consistency (alpha = 0.75) and test-retest reliability (0.72) [27].

### Statistical analyses

Results of participants who completed the assessments (*n* = 1591) were weighted up to project the total number of individuals in the different research sites and the data were further weighted to make sure that the sociodemographic distribution (i.e. gender, age, urban vs. rural) of the sample corresponded to population census data. Several analyses were done. First, cross-tabs were used to gain insight into the distributions of the responses to the individual items of the PDD and MHKQ. Second, PDD and MHK scores were compared between sociodemographic groups, using independent samples t-tests or one-way ANOVA. Third, to investigate the association between stigma (subdomains) and MHK, univariate linear regression analyses were run with stigma score as dependent variable and MHK score as independent variable. Finally, these analyses were run with covariates (sociodemographic factors and experienced general psychological distress). The analyses were run with SPSS (v19.0) with alpha set at 0.05 (two tailed).

## Results

### Sample characteristics

Socio-demographic characteristics are shown in Table 1.

**Table 1:** Socio-demographic characteristics of the participants (*n* = 1591)

Variables	Unweighted(%)	Weighted (%)
Sex		
female	887(55.8)	741(46.6)
male	704(44.2)	850(53.4)
Resident area		
urban	1173(73.7)	1288(81.0)
rural	418(26.3)	303(19.0)
Age		
18–39	446(28.0)	784(49.3)
40–54	493(31.0)	425(26.7)
55+	652(41.0)	382(24.0)
Education, year		
0–6	381(23.9)	239(15.0)
7–9	532(33.4)	470(29.6)
10–12	371(23.3)	421(26.5)
13+	307(19.3)	461(29.0)
Pre capital family income		
above median	751(47.2)	713 (44.8)
below median	840(52.8)	878(55.2)
Employment status		
housewife	145(9.1)	176(11.0)
having a work	659(41.4)	862(54.2)
retired	474(29.8)	268(16.9)
jobless or lose of job	166(10.4)	170(10.7)
famer	159(10.0)	114(7.2)
Marital status		
never married	114(7.2)	279(17.6)
married	1268(79.7)	1277(77.1)
divorce/lose spouse	209(13.1)	85(5.3)
GHQ score		
≥ 4	1494(93.9)	1490(93.7)
< 4	97(6.1)	101(6.3)

### Stigma

The PDD item responses are shown in Table 2. To statements about devaluation, a considerable number of participants (9.2–14.5%) responded that they were ‘not sure’. Of those providing a response, the majority agreed that others see persons with mental illness as just as intelligent (item 2; 47.7%) and trustworthy (item 3; 47.0%) as others, and disagreed that others see entering a mental hospital as a personal failure (item 5; 69.2%) or think less of persons that enter a mental hospital (item 7; 62.4%). However, a majority of participants agreed with the statement that most people will take a person’s opinion less serious once they know a person has been in a mental hospital (item 12; 55.3%).

**Table 2:** The public’s perception of the stigma attached to former mental patients in Tianjin^a^

Items	Agree (%)	Not sure (%)	Disagree (%)
1. Most people would willingly accept a former mental patient as a close friend	43.3	11.7	44.9
2. Most people believe that a person who has been in a mental hospital is just as intelligent as the average person	47.7	13.0	39.3
3. Most people believe that a former mental patient is just as trustworthy as the average citizen	47.0	13.9	39.1
4. Most people would accept a fully recovered former mental patient as a teacher of young children in a public school	31.9	13.0	55.1
5. Most people feel that entering a mental hospital is a sign of personal failure (R)	16.0	14.8	69.2
6. Most people would not hire a former mental patient to take care of their children, even if he or she had been well for some time (R)	68.2	8.2	23.6
7. Most people think less of a person who has been in a mental hospital (R)	28.4	9.2	62.4
8. Most employers will hire a former mental patient if he or she is qualified for the job	43.7	17.0	39.3
9. Most employers will pass over the application of a former mental patient in favor of another applicant (R)	54.9	13.6	31.5
10. Most people in my community would treat a former mental patient just as they could treat anyone	65.7	16.7	17.5
11. Most young women would be reluctant to date a man who has been hospitalized for a serious mental disorder (R)	70.6	14.0	15.4
12. Once they know a person was in a mental hospital, most people will take his opinions less seriously (R)	55.3	14.5	30.2

Note: Respondents who endorsed the two points on either side of the mid-point of the five-point scales (values 1 + 2 and 4 + 5) were grouped together to the categories ‘agree’ and ‘disagree’

*R* Reversed item;

^a^Percentages were calculated after weighting

To statements about discrimination, a sizable percentage (11.7–17.0%) responded that there were ‘not sure’. Of the other participants, the majority disagreed with the statement that a former mental patient would be hired as a teacher (item 4; 55.1%) and agreed with the statements that most people would not hire a (former) mental patient to take care of their kids (item 6; 68.2%), that most employers would pass over the application of a former mental patient (item 9; 54.9%), and that most women would be reluctant to date a man with a history of mental illness (item 11; 70.6%). However, roughly equal percentages of participants agreed and disagreed with the statement that most employers will hire former mental patient if he/she is qualified for the job (item 8; 43.7% agree vs. 39.3% disagree) and the statement that most people would willingly accept a former mental patient as a close friend (item 1; 43.3% agree vs. 44.9% disagree). The majority agreed with the statement that most people in their community would treat a former mental patient just as they would treat anyone (item 10; 65.7%).

Mean devaluation scores were higher in rural compared to urban areas, older age groups, those with 0–6 years of education, those with below-median income and farmers (See Table 3). Discrimination scores showed no differences across sociodemographic groups.

**Table 3:** Socio-demographic characteristics and differences in scores of PDD scale and MHKQ by socio-demographic variables

Variables	Devaluation		Discrimination		MHK	
	Mean ± sd	t/F, P	Mean ± sd	t/F, P	Mean ± sd	t/F, P
Sex						
female	13.9 ± 3.1	0.08, 0.94	22.5 ± 4.3	0.55, 0.58	11.6 ± 1.9	−1.61, 0.11
male	13.9 ± 3.3		22.4 ± 4.5		11.7 ± 2.2	
Resident area						
urban	13.8 ± 3.1	**1.99, 0.05**	22.5 ± 4.3	045, 0.66	11.8 ± 2.0	**5.46, < 0.001**
rural	14.3 ± 3.5		22.4 ± 4.5		11.2 ± 1.9	
Age						
18–39	13.6 ± 3.0	**7.58, 0.006**	22.5 ± 4.4	2.67, 0.10	12.1 ± 2.1	**26.49, < 0.001**
40–54	14.0 ± 3.4		22.6 ± 4.1		11.6 ± 2.1	
55+	14.4 ± 3.3		22.1 ± 4.8		11.2 ± 1.8	
Education, year						
0–6	15.0 ± 3.6	**21.39, < 0.001**	23.0 ± 4.6	0.75,0.39	10.7 ± 1.5	**130.38, < 0.001**
7–9	13.8 ± 3.1		22.1 ± 4.2		11.4 ± 1.8	
9–12	13.6 ± 3.3		21.9 ± 4.5		11.9 ± 2.1	
13+	13.7 ± 3.0		23.0 ± 4.2		12.4 ± 2.2	
Per capita family income						
above median	14.0 ± 3.2	**2.22, 0.03**	22.6 ± 4.3	0.67, 0.50	12.0 ± 2.2	**4.61, < 0.001**
below median	14.4 ± 3.3		22.7 ± 4.3		11.5 ± 1.9	
Employment status						
housewife	13.1 ± 3.2	**14.18, < 0.001**	21.2 ± 4.3	0.01, 0.94	12.1 ± 2.1	**47.49, < 0.001**
having a job	13.9 ± 3.2		22.8 ± 4.4		11.9 ± 2.1	
retired	14.2 ± 3.1		22.4 ± 4.2		11.4 ± 1.9	
jobless or lost job	14.1 ± 3.2		22.1 ± 4.4		11.6 ± 1.7	
farmer	14.5 ± 3.7		22.2 ± 4.5		10.6 ± 1.7	
Marital status						
never married	13.8 ± 3.0	8.03, 0.24	22.3 ± 4.1	0.67, 0.41	12.1 ± 2.2	**18.67, < 0.001**
married	13.9 ± 3.3		22.5 ± 4.5		11.7 ± 2.0	
divorced/lost spouse	14.4 ± 3.3		22.8 ± 4.0		11.1 ± 2.1	
GHQ score						
≥ 4	14.2 ± 3.4	0.82, 0.411	21.9 ± 4.3	1.25, 0.210	12.9 ± 2.5	1.16, 0.248
<4	13.9 ± 3.2		22.5 ± 4.4		13.2 ± 2.6	

Bold values significance level was set at 0.05; two-tailed test

### Mental health knowledge

The responses on the MHK items are shown in Table 4. High percentages of participants showed signs of basic knowledge about mental health, as shown by their affirmative responses (all > 80%) to statements about mental health as an integral part of health (item 1), everyday aspects that are helpful to keep good mental health (item 11), the unnoticed existence of mental health problems (item 3), the existence of multiple components of mental health (item 5), the possibility of mental-health problems at any age (item 8), the role of stress and/or major life events in mental health problems (item 16), the need to see a mental health professional in case of mental problems (item 7), and the heritability of mental health problems (item 12). Also, 75.2% acknowledged the fact that mental problems can influence adolescents’ functioning (item 13). There were also signs of lacking knowledge in the population: 48.7% of respondents thought middle-aged or older adults were less likely to have mental problems (item 14), 43.2% believed that mental health problems cannot be cured (item 6), 59.4% agreed that mental illness cannot be prevented (item 9), and 46.0% agreed that diagnosed patients should only take medication for a short period of time (item 10). Of the respondents, 86.7% agreed with the statement that persons with an unstable temperament were more likely to have mental illness (item 15), 75.0% endorsed the statement that mental illness results from something wrong in thought (item 2) and 69.7% endorsed the statement that all mental illness is caused by stress (item 4).

**Table 4:** The public’s knowledge related to mental health in Tianjin

Items	yes	no
1. Mental health is an integral part of health.	99.0	1.0
2. Mental illnesses result from something wrong in thought.	75.0	25.0
3. Most people may have a mental problem, but they may not notice the problem.	97.7	2.3
4. Mental illnesses are all caused by stress.	69.7	30.3
5. Mental health includes normal intelligence, stable mood, harmonious relationships, and good ability to adapt and so on.	95.6	4.4
6. Most mental illnesses cannot be cured.	43.2	56.8
7. If you suspect that you have mental problems or mental illnesses, you should go to a psychiatrist or psychologist for help.	89.9	10.1
8. Individuals at any age can have a mental problem.	95.4	4.6
9. Mental illnesses or psychological problems cannot be prevented.	59.4	40.6
10. Even though a person is diagnosed with a severe mental disorder, he/she should take medication for only a short period rather than continuously for a long term.	46.0	54..0
11. An optimistic attitude towards life, good interpersonal relationships and a healthy lifestyle are helpful to keep a good mental health.	98.2	1.8
12. Persons with a family history of mental disorders have a higher chance to develop mental disorders or mental problems.	82.1	17.9
13. Mental problems in adolescents do not influence their academic achievement.	24.8	75.2
14. It is less likely to have mental problems or disorders in middle or old age.	48.7	51.3
15. Someone with an unstable temperament is more prone to have mental problems.	86.7	13.3
16. High psychological stress or major life events could induce mental problems or disorders.	94.4	5.6

MHK levels were lower in rural areas, older age-groups, the low (0–6 years) education group, farmers, and in those who were never married (Table 3).

### The association between MHK and stigma

In univariable analyses (Table 5), devaluation, discrimination and PDD total score all showed significant negative associations with MHK score (R^2^ = 0.01–0.07), with the strongest association between MHK and devaluation (R^2^ = 0.07). When adjusted, the associations remained largely the same (R^2^ = 0.04–0.10).

**Table 5:** unadjusted and adjusted associations between MHK and public stigma

	Stigma outcomes					
	PDD devaluation			PDD discrimination		
	B (95%CI)	Beta	R^2^	B (95%CI)	Beta	R^2^
MHK unadjusted model	−0.425	−0.271	0.074	−0.22	−0.103	0.011
	(−0.499 - -0.351)*			(− 0.324 - -0.116)*		
MHK	−0.389	−0.248	0.095	−0.24	− 0.113	0.041
adjusted model	(−0.466 - -0.311)*			(−0.348 - -0.131)*		

In the adjusted model the reported coefficient is adjusted for the following dichotomous/categorical sociodemographic covariates: gender, rural-urban, age-group, education-group, income-group, employment status, marital status and GHQ score. *p < 0.001

## Discussion

This study showed that the Tianjin public holds some negative attitudes to mental health patients, especially with regard to engaging in closer personal relationships with (former) mental patients. Most people did have general knowledge about how to obtain and maintain mental health, but had less knowledge about the causes, treatment and prevention of mental illness. MHK level was negatively associated with devaluation and discrimination of persons with mental health problems. When asked about others’ perceptions of (former) mental patients, many participants thought that the public holds a negative attitude with regard to engaging in closer personal relationships with (former) mental patients. These findings are in line with previous studies in China [10, 13, 28, 29]. Interestingly, negative attitudes were more often endorsed on discrimination items than on devaluation items, indicating that public stigma may be especially manifested in discrimination in the closer personal realm.

### Limitations of this study

The present study has several limitations. First, the MHK questionnaire was developed by the Chinese MOH for use in China specifically, limiting generalizability of the findings to other regions of the world. However, several well-known aspects of MHK [30] were included in the MHK questionnaire and several items were quite similar to those used in other developed scales [31–33] and used in other studies [34, 35]. Second, the generalizability of the findings to all of China may be limited due to large regional differences. However, the Tianjin area is quite a typical example of the highly urbanized areas, of which many are found in China and that make up a large part of its population. Third, the cross-sectional nature of the study forbids any causal inference.

### Public stigma on mental illness

The PDD has been used to investigate mental health stigma in many other countries, such as Germany [36], Vietnam [37], Russia and Slovakia [38]. On some items, the Tianjin public seemed to endorse more negative attitudes than other investigated populations. For example, 47.7% of the Tianjin sample stated that most others thought a person who has been in a mental hospital is just as intelligent as the average person, compared to percentages that ranged from 49.3% (Vietnam) to 67.8% (Russia). However, 69.2% of the Tianjin sample reported that they did not think that most people see entering a mental hospital as a sign of personal failure, compared to percentages in other samples that ranged from of 32.0% (Germany) to 51.9% (Russia). Also, 62.4% of the Tianjin sample stated that they did not think that most others thought less of a person who has been in a mental hospital compared to percentages of 21.8% (Germany) to 44.8% (Vietnam). With regard to the PDD discrimination items, The Tianjin sample showed a more positive attitude toward the job opportunities of former mental patients even being a teacher in a public school: 43.7% agreed that most employers will hire a former mental patient, compared to percentages in other samples that ranged from 23.3% (Slovakia) to 40.6% (Vietnam); 31.9% of the Tianjin sample agreed with the statement that most people would accept a fully recovered former mental patient as a teacher of young children in a public school, which was higher than in Slovakia and Germany (15.5 and 27.6%, respectively). Also, a majority of the Tianjin sample stated that they thought that most persons treat a former mental patient like they would treat anyone, compared to percentages in other countries from 28.3% (Slovakia) to 46.8% (Vietnam). However, when circumstances were related to close relationships (being a close friend with a former mental patient, letting a former mental patient take care of their children or dating a former mental patient), the Tianjin sample showed a higher endorsement of statements about rejection of (former) mental health patients. For example, 44.9% of Tianjin sample disagreed with the statement that people would accept a former mental patient as a close friend, compared to lower percentages in in Russia (24.7%), Slovakia (29.6%), Germany (23.9%) and Vietnam (25.9%). This results were consistent with the results in the Global Context–Mental Health Study, which showed that the highest levels of stigmatizing responses were concentrated on aspects relating to providing child care, marriage into the family and teaching of children [6]. The survey in Beijing [13] and a survey using a web-based approach [12] also have shown that while most respondents perceived the public has positive attitude about the status of individuals with mental illness, they perceived more negative attitudes when considering personal interactions. More previous studies have shown that discrimination prominently occurred in work, marriage, and interpersonal relationships which are closely related to basic social life [10, 39].

The observed attitudes toward mental illness and differences from other countries could be related to cultural factors [29, 38, 40]. Yang et al. [41, 42] showed that the particular manifestations of stigma in Chinese people are shaped by cultural meanings embedded in Confucianism, pejorative etiological beliefs about mental illnesses and the centrality of ‘face’ (i.e. one’s moral standing within society. One of the central principles of Confucianism is that every member of society must follow the moral demands of society to achieve personal and social harmony. People with mental illnesses may be unable to fully meet these demands, leading others to question their moral status. In addition, Chinese traditional beliefs attribute mental illness to possession by demons, wrongful child bearing behavior, or wrong-doing by one’s ancestors [40]. As a result, people with psychiatric illnesses are regarded as ‘morally bankrupt’ and relegated to a lower moral level. Losing one’s moral standing or ‘face’ in a community may lead others to avoid contact out of fear for moral contamination, leading to social exclusion (‘social death’) [42]. Patients may be excluded from participating in social networks to prevent embarrassment to the family and lowering of their moral status. These specific Chinese cultural factors could explain why manifestations of public stigma toward mental illness are observed especially in closer personal relationships. However, the results also showed relatively positive public attitudes with regard to mental illness patients in general, which could partly be due to previous efforts to reduce public stigma, although this cannot be determined based on the current study.

### Mental health knowledge

The MHK results showed that, on the one hand, most people did have basic knowledge about how to obtain and maintain mental health: above 90% of individuals had correct general knowledge mental health. On the other hand, more specific knowledge, for instance about the manifestations of mental illness in different age periods and the influences of mental illness on adolescents, was less widespread. A similar ambiguity could be observed in the reported knowledge about the causes of mental illness: most people thought the family history and ‘unstable temperament’ were risk factors to develop mental disorders, but only about 70% believed that mental illness could result from something wrong in thought or stress. Above 90% of the respondents knew that seeking professional help was necessary in case of mental illness but many (43%) also thought mental illness could not be cured or prevented. The same MHK questionnaire has previously been administered in Zhengzhou and Guangzhou, where similar response patterns were found [43, 44]. About 50% people think that it is less likely to have mental problems or disorders in middle or old age. An explanation might be that general public might not see dementia as a psychiatric illness. Another explanation might be that people at middle or old age might be perceived to be at a low risk to develop a mental disorder because of having a ‘stable temperament’. There have also been studies comparing MHK between China and other countries. A study comparing Chinese and British beliefs about schizophrenia showed that Chinese respondents held more religious and superstitious beliefs and British respondents put more emphasis on beliefs about causes and treatment [34]. Two other studies [16, 17] on MHK in Chinese subjects in Shanghai, Chinese-speaking Australians in Melbourne and Chinese subjects in Hong Kong showed lower MHK in Shanghai Chinese than Hong Kong and Australian Chinese. Another nationally-representative survey in China to understand the public’s profile of mental health literacy showed a low recognition of mental health problems and that this recognition was better for depression than schizophrenia [14]. Together, these results suggest that specific knowledge about mental illness in the Chinese population is relatively limited. This specific knowledge could be a particular focus of attention in future educational campaigns. The current results suggest that such campaigns could be specifically targeted at socioeconomic groups with comparatively lower MHK, including rural residents, older persons (> 39 years), those with no/low education and farmers.

### The relationship between stigma and MHK

The results showed that MHK was negatively associated with devaluation and discrimination, in line with previous findings [45] and supporting the idea that improving MHK could help reduce public stigma. However, there have also been studies that found no or an inverse relationship between knowledge-levels and attitudes towards people with mental illness [15]. For instance, a longitudinal study in Germany showed that an increase of public knowledge did not change or even increased the desire of subjects for social distance from people with mental illness [36]. A review of studies about mass media intervention, a common way to increase MHK and to reduce prejudice and discrimination, concluded that mass media interventions may reduce prejudice, but that there was insufficient evidence to determine the effects on discrimination [46] . Reviews of the literature on interventions to reduce mental-health stigma in the medium and long term, found some evidence for the effectiveness of such interventions [47, 48]. Future research is required to evaluate the use of smartphone applications to increase MHK [49] and support caregivers [50]. Taken together, the current and previous results indicate that increased MHK may be related to less prejudice, devaluation and discrimination of mental-health patients. However, the extent to which MHK can actually be modified by interventions and whether such changes in MHK are causally related to decreases in public stigma remains a topic for further study.

## Conclusions

In conclusion, there is room for improvement with regard to levels of public stigma and MHK in Tianjin. The observed negative association between stigma and MHK indicates that, in addition to targeted programs to reduce mental health stigma, improvement of public MHK could contribute to reduction of public stigma.

## Data Availability

All the data supporting our findings have been presented in the manuscript; the datasets used and/or analyzed during the current study are available from the corresponding author on reasonable request.

## Abbreviations

GHQ-12: 12-item General Health Questionnaire
MHK: Mental Health Knowledge
MHKQ: Mental Health Knowledge Questionnaire
NOS: Not Otherwise Specified
PDD: The Perceived Discrimination and Devaluation scale
SCID: The Structured Clinical Interview for DSM-IV axis I disorders
TJMHS: The Tianjin Mental Health Survey
